# Efficient Extraction and Antioxidant Capacity of Mycosporine-Like Amino Acids from Red Alga Dulse *Palmaria* *palmata* in Japan

**DOI:** 10.3390/md18100502

**Published:** 2020-09-30

**Authors:** Yuki Nishida, Yuya Kumagai, Shunta Michiba, Hajime Yasui, Hideki Kishimura

**Affiliations:** 1 Chair of Marine Chemical Resource Development, Graduate School of Fisheries Sciences, Hokkaido University, Hakodate, Hokkaido 041-8611, Japan; karakuchi@eis.hokudai.ac.jp (Y.N.); shun_ta_soft@yahoo.co.jp (S.M.); 2 Laboratory of Marine Chemical Resource Development, Faculty of Fisheries Sciences, Hokkaido University, Hakodate, Hokkaido 041-8611, Japan; yuyakumagai@fish.hokudai.ac.jp; 3 Laboratory of Humans and the Ocean, Faculty of Fisheries Sciences, Hokkaido University, Hakodate, Hokkaido 041-8611, Japan; hagime@fish.hokudai.ac.jp

**Keywords:** red alga, dulse, mycosporine-like amino acids, monthly variation, antioxidant capacity

## Abstract

Mycosporine-like amino acids (MAAs) are the ultraviolet (UV)-absorbable compounds, which are naturally produced by cyanobacteria and algae. Not only these algae but also marine organisms utilize MAAs to protect their DNA from UV-induced damage. On the other hand, the content of MAAs in algae was changed by the environmental condition and season. In addition to the UV-protected function, the antioxidant capacity of MAAs can apply to the cosmetic sunscreen materials and anti-cancer for human health. In this study, we developed the efficient extraction method of MAAs from red alga dulse in Usujiri (Hokkaido, Japan) and investigated the monthly variation. We also evaluated the antioxidant capacity. We employed the successive extraction method of water and then methanol extraction. Spectrophotometric and HPLC analyses revealed that the yield of MAAs by 6 h water extraction was the highest among the tested conditions, and the content of MAAs in the sample of February was the most (6.930 µmol g^−1^ dry weight) among the sample from January to May in 2019. Antioxidant capacity of MAAs such as crude MAAs, the purified palythine and porphyra-334 were determined by 2,2’-azinobis(3-ethylbenzothiazoline 6-sulfonic acid) (ABTS) radical scavenging and ferrous reducing power assays in various pH conditions, showing that the highest scavenging activity and reducing power were found at alkaline condition (pH 8.0).

## 1. Introduction

Ultraviolet radiation (UVR) is a part of the solar electromagnetic spectrum and defined as wavelengths from 200 to 400 nm composed of ultraviolet A (UVA) (315–400 nm), ultraviolet B (UVB) (280–315 nm), and ultraviolet C (UVC) (200–280 nm). UVR reaching on the Earth’s surface is only a small portion of the entire UVR, which is composed of wavelengths above 290 nm (mainly UVA and up to 10% of UVB) [1,2,3]. In the past few decades, the amount of UVR on the Earth’s surface has been increased due to climate change with the decrease in aerosols and cloud [4,5], resulting in exposure of UVR to a wide variety of biological systems. In human, exposure of UVR induces diseases such as cancer and skin aging by denature of DNA or proteins (e.g., formation of cyclobutane purine/pyrimidine dimers) [6,7]. Therefore, organisms under UVR must take some defense strategies to minimize UV-induced damage [1,8].

Mycosporine-like amino acids (MAAs) are synthesized and accumulated as photoprotective compounds by marine phototrophs (e.g., dinoflagellates, cyanobacteria and macro algae) [1,9,10]. MAAs are nitrogenous secondary metabolites of the low molecular weight (<400 Da) with the maximum absorption ranging from 310 to 360 nm and have the high molar extinction coefficients (28,100 to 50,000 M^−1^ cm^−1^) [10,11]. MAAs are composed of a cyclohexanone or cyclohexenimine ring conjugated with amino acids, amino alcohols, or amino groups. MAAs are classified into two types by the core structure of cyclohexanone or cyclohexenimine ring, showing oxo-MAAs or imino-MAAs, respectively. The major oxo-MAAs are mycosporine-glycine and mycosporine-taurine, while major imino-MAAs are shinorine, palythine, asterina-330, porphyra-334, usujirene and palythene [1,10]. According to Hoyer et al. [12], the biosynthesis pattern of MAAs in red alga is classified into three categories: (i) species with no capacity for MAAs biosynthesis; (ii) species with the high content of MAAs permanently; (iii) species changing the content of MAAs with the environmental conditions. While the species of category (i) typically represent red seaweed of lower sublittoral species of category (ii) and (iii) grow from the mid-sublittoral zone to the supralittoral zone [12,13]. Biosynthesis of MAAs is expected to the shikimate pathway and the pentose phosphate pathway, and many MAAs are produced in the synthesis steps [1,10,14,15,16,17]. However, the ratio and product patterns of MAAs are still unclear because of the complicated environmental condition and the lack of knowledge for seaweed’s molecular biology [18].

MAAs have the physicochemical advantage in the point of high melting point, photostability, solubility in water, and organic solvents, stability in a wide range of pH and temperature [9,11,14,19,20,21]. UV-protective compounds are divided into two types: UV-reflective and UV-absorbable. MAAs are UV-absorbable materials and dissipate the energy as heat without generating reactive oxygen species (ROS) [9,10,13,22]. Recently, functions of MAAs have been reported as sunscreens, activators of cells proliferation, anti-cancer agents, anti-photoaging molecules, and stimulators of skin renewal. MAAs are nontoxic and biodegradable compounds. Therefore, MAAs have been attracted attention among various biotechnological industries [1,9,11]. Antioxidant capacity is one of the main functions of MAAs [1,10,21,23]. The in vitro antioxidant capacity has been reported using oxygen radical absorbance capacity (ORAC) assay (measuring hydrogen atom transfer (HAT) reaction) and 2,2′-azino-bis (ABTS) radical scavenging assay (measuring electron transfer (ET) reaction) [10,21,23,24,25]. On the other hand, inconsistencies in strength of antioxidant capacity remain an issue [24,25]. Although research on relationship between the activity and pH has been reported [10,21], antioxidant capacity of MAAs is still controversial.

Among macro algae, dulse (*Palmaria palmata*) is known to possess MAAs [18,24]. Dulse is a red alga distributed mainly across coastal areas in high-latitude such as Ireland and Atlantic Canada. Dulse also grows well in Hokkaido, Japan. Hakodate, the southern part of Hokkaido, is famous for Kombu farming, and dulse grew there and disturbed growth of Kombu (*Laminaria* sp.) [26]. Dulse is underused alga in Japan; therefore, the use of dulse was requested. To utilize dulse in Usujiri, Hakodate, Japan, we clarified genetic character [27,28] and showed the nutrient characters (approximately 40 g protein/100 g dried dulse). The major component of proteins is phycoerythrin (PE) [29], and the thermolysin hydrolysate showed the angiotensin-I-converting enzyme inhibitory activity [26,30,31]. The chromophores from PE showed antioxidant capacity [32]. In addition, β-(1→3)-xylosyl-xylobiose prepared from dulse xylan exhibited the prebiotic effect on enteric bacterium [33]. In this way, dulse contains many healthy functional ingredients. On the other hand, seaweeds contain low content of MAAs (up to 14 mg g^−1^ dry weight) [9,13]. Usujiri dulse grew in the depth of 1–2 m, which is expected a large content of MAAs, since the depth of biotope was a key factor of MAAs synthesis [12]. In order to use MAAs from Usujiri dulse for biotechnological applications, it is necessary to develop the efficient extraction method. However, little has been reported on the efficient extraction from macro algae.

In this study, we first determined the efficient extraction condition to evaluate the content of MAAs of Usujiri dulse and then investigated the monthly variation (January–May 2019). Finally, we evaluated the effects of pH on the antioxidant capacity of MAAs by ABTS radical scavenging and ferrous reducing power assays.

## 2. Results

### 2.1. Determination of Extraction Conditions of Dulse MAAs

**Figure 1:**
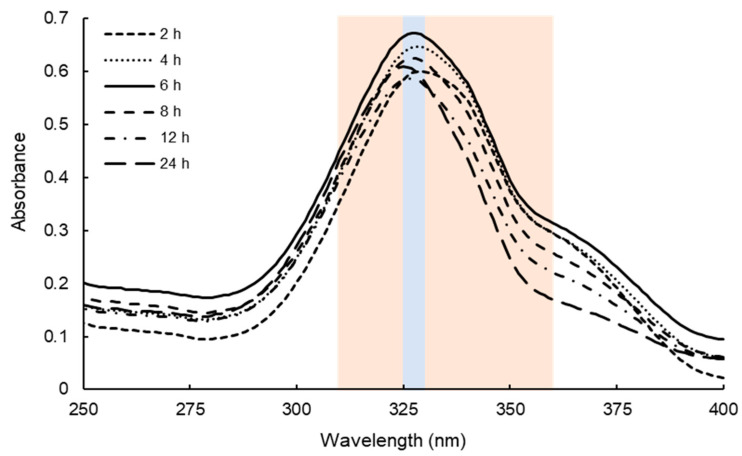
Absorption spectra of dulse crude MAAs solutions. Dulse crude MAAs solutions were prepared by different water extraction terms (2 to 24 h) using the sample collected on 13 March 2018. All six solutions were diluted with 20 volumes (*v/v*) of distilled water. Blue and orange backgrounds represent the absorption maximum (325–330 nm) and the area for calculation of MAAs (310–360 nm), respectively.

Although MAAs have been extracted from various kinds of macro and micro algae, the extraction term and condition differed in papers [18,23]. Our preliminary experiments revealed that the water extraction term drastically affected the MAAs yields. Therefore, we attempted to determine the water extraction term of MAAs. Usujiri dulse, which was collected on 13 March 2018, was employed to determine the condition, and water extraction term was investigated for 2 to 24 h. The absorption spectra of dulse crude MAAs solution are shown in Figure 1. The absorption maximum peaks of the six solutions were detected ranging from 325 to 330 nm. The content of MAAs was calculated by integration of absorbance from 310 to 360 nm where MAAs exhibit the absorption maximum. As a result, the solution prepared by water extraction for 6 h showed the highest absorbance as compared with the others.

**Figure 2:**
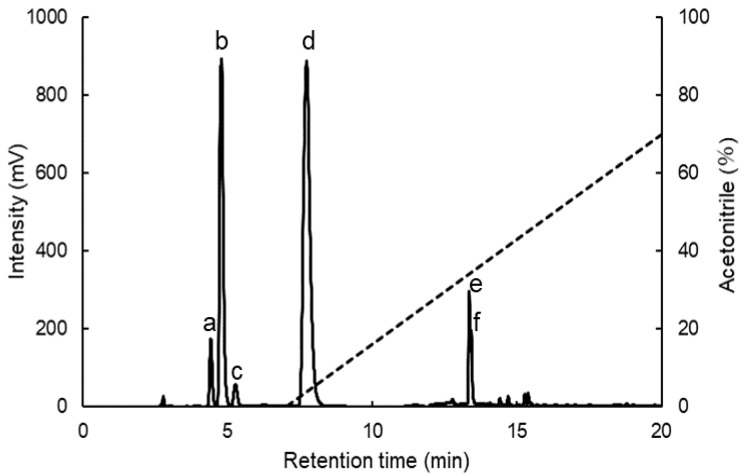
HPLC chromatogram of dulse crude MAAs solution. The data represents 6 h sample showing the typical peaks at retention time of 4.42 min (**a**), 4.79 min (**b**), 5.27 min (**c**), 7.73 min (**d**), 13.36 min (**e**), and 13.43 min (**f**).

We then clarified the components of MAAs in Usujiri dulse by high-performance liquid chromatography (HPLC) analysis. Figure 2 shows the chromatogram of dulse crude MAAs solution (6 h). HPLC analysis revealed the presence of six prominent peaks (Peaks a–f) at 4.42, 4.79, 5.27, 7.73, 13.36 and 13.43 min with the absorption maxima (λ_max_) at 329–331, 319, 325–328, 331, 357, and 358 nm, respectively. To determine the mass of each MAA, the MAAs fractions were collected and subjected to MALDI-TOF/MS analysis. The HPLC-purified MAAs (Peaks a–f) showed a prominent ion peak of protonated molecules ([M + H]^+^) at *m*/*z* 333.2, 245.1, 289.2, 347.1, 285.1, and 285.1, respectively. Based on UV-Vis-absorption spectra and MALDI-TOF/MS analysis, the components of MAAs (Peaks a–f) in Usujiri dulse were identified as shinorine (Peak a; λ_max_: 329–331 nm; *m*/*z* 333.2), palythine (Peak b; λ_max_: 319 nm; *m*/*z* 245.1), asterina-330 (Peak c; λ_max_: 325–328 nm; *m*/*z* 289.2), porphyra-334 (Peak d; λ_max_: 331 nm; *m*/*z* 347.1), usujirene (Peak e; λ_max_: 357 nm; *m*/*z* 285.1), and palythene (Peak f; λ_max_: 358 nm; *m*/*z* 285.1) (Table 1).

We identified six major MAAs (Peaks a–f in HPLC) by HPLC and MALDI-TOF/MS analyses. Using Lambert–Beer law, we prepared a standard curve for each MAA in HPLC peak area and then estimated the content of each MAA by HPLC. The peaks, usujirene and palythene, were not separated by HPLC, but their extinction coefficient was quite similar (Table 1). Therefore, we expressed these MAAs as usujirene + palythene and employed the high extinction coefficient from palythene to avoid the risk of overestimation. Table 2 shows the content of MAAs from dulse at each water extraction term (2, 6, and 24 h). Palythine and porphyra-334 were the major components of Usujiri dulse and occupied more than 90% of total MAAs. Water extraction term did not affect the components of MAAs. There was a significant difference (*p* < 0.05) in the content of MAAs extracted between 2 and 6 h, and at 2 and 24 h, respectively. Only the content of palythine was increased in the extraction term. We will discuss the reason later in discussion section. The content of MAAs was maximized by 6 h extraction, which was higher than that by 2 h (1.13-fold) and 24 h (1.04-fold). Therefore, we determined the water extraction for 6 h in the following experiments.

### 2.2. Monthly Variation of MAAs

In the above section, we clarified the importance of water extraction term for MAAs and prepared the standard curve for HPLC analysis. In this section, we investigated the monthly variation of MAAs using samples collected on 23 January, 25 February, 2 April, and 17 May 2019. Dulse crude MAAs solutions were prepared by water extraction for 6 h, and the absorption spectra were measured. The absorption maximum peak of the four solutions was in the range of 328 to 339 nm. The content of MAAs was estimated by integration in the range of 310 to 360 nm. As a result, the sample of 25 February showed the highest absorbance (6.930 µmol g^−1^ DW), while that of 23 January exhibited the lowest absorbance (2.649 µmol g^−1^ DW) (Table 3). There was a significant difference (*p* < 0.05) in the content of MAAs among each month. The monthly variation of the content of MAAs was increased sharply from January to February, stable from February to April, and gradually decreased from April to May.

**Figure 3:**
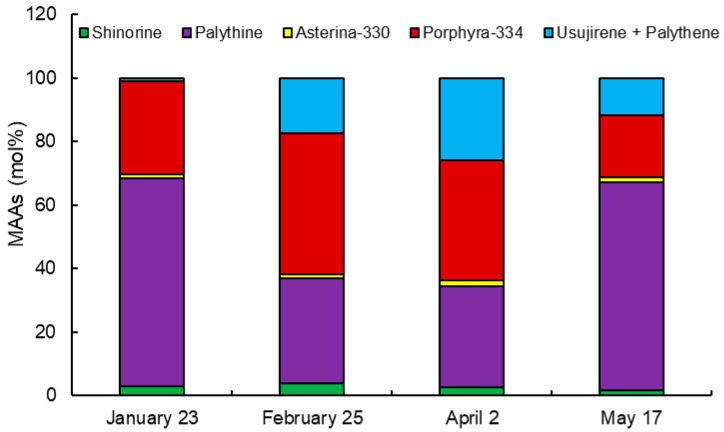
Molar percentage of MAAs in 2019. The content of six MAAs, shinorine, palythine, asterina-330, porphyra-334 and usujirene + palythene, was compared in dulse collected on 23 January, 25 February, 2 April and 17 May. The data showed mean values, *n* = 3.

The molar percentage (mol%) of the content of each MAA in 2019 is shown in Figure 3. The mol% of shinorine (from 1.8 mol% on 17 May to 3.8 mol% on 25 February) and asterina-330 (from 1.3 mol% on January 23 to 1.8 mol% on 2 April) was stable at low values. Palythine, showing the high mol% on 23 January and 17 May, varied from 65 to 33 and 32 mol% on 25 February and 2 April, respectively. On the other hand, porphyra-334 and usujirene + palythene were low mol% on 23 January and 17 May. The mol% of these MAAs was increased on 25 February and 2 April, especially usujirene + palythene were 29-folds increase from 0.9 mol% on 23 to 26 January mol% on 2 April.

### 2.3. ABTS Radical Scavenging Activity of Dulse MAAs

Antioxidant capacity is one of the main functions of MAAs. Despite many studies, research on the activity related to pH was limited. Torres et al. [10] reported that high antioxidant capacity of imino-MAAs (shinorine and porphyra-334) was found under alkaline condition and ABTS radical scavenging activity of palythine was higher than that of shinorine and porphyra-334. However, effect of pH on palythine was not reported. ABTS radical scavenging assay is based on Reaction 1 (ABTS•^+^ and MAA• represent ABTS radical cation and MAA radicals, respectively) [37,38]:

ABTS•^+^ + MAA-H → ABTS + MAA• + H^+^ (1)

**Figure 4:**
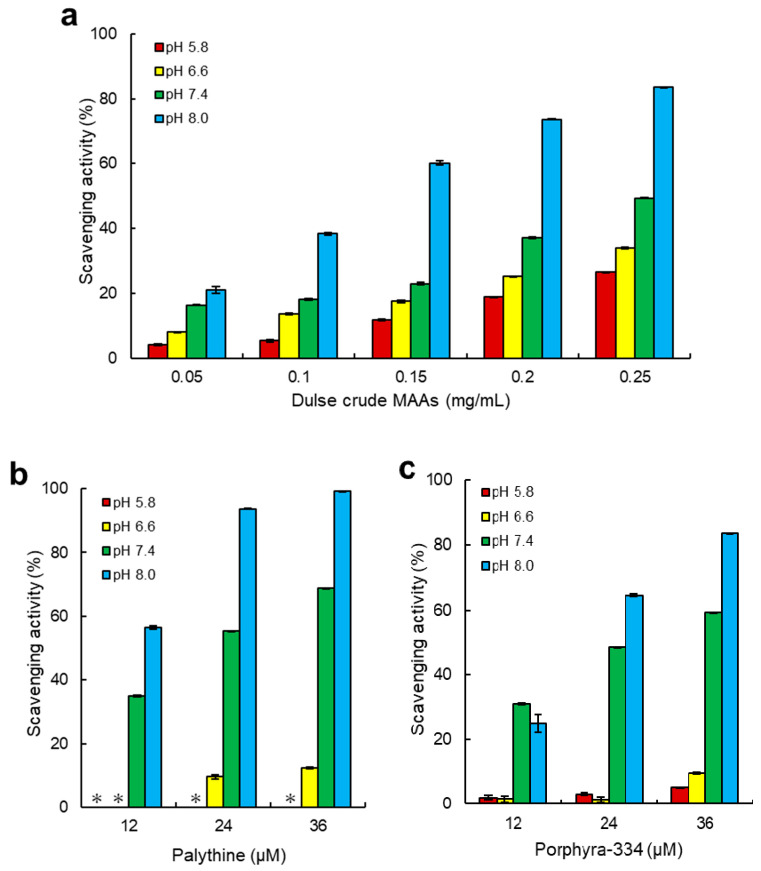
Antioxidant scavenging activity. (**a**), dulse crude MAAs; (**b**), palythine; (**c**), porphyra-334. ABTS radical scavenging assay was performed at pH 5.8, 6.6, 7.4 and 8.0. Data represent mean values ± SE (*n* = 3). An asterisk (*) indicates the activity was limit of detection. The components of dulse crude MAAs in 0.15 mg/mL was as follows: shinorine (0.3 µM), palythine (2.4 µM), asterina-330 (0.1 µM), porphyra-334 (3.2 µM) and usujirene + palythene (1.3 µM).

The main components of MAAs in Usujiri dulse were palythine and porphyra-334. Therefore, we evaluated pH dependence of ABTS radical scavenging activity using dulse crude MAAs, palythine and porphyra-334, showing the activity in the tested samples (Figure 4). Among measured pH, the antioxidant capacity was increased in the alkaline condition, and the maximum radical scavenging activity was at pH 8.0. The IC_50_ (the content of MAAs that reduce half of the maximal response of ABTS radical scavenging activity) values of dulse crude MAAs at pH 5.8, 6.6, 7.4, and 8.0 were 0.36, 0.33, 0.28, and 0.14 mg/mL, respectively (Table 4). Palythine and porphyra-334 also showed the highest ABTS radical scavenging activity at alkaline condition (pH 8.0) with the IC_50_ values of 12.0 and 20.8 µM, respectively. The IC_50_ value of ascorbic acid, which is known to be an excellent scavenger, was 8.9 µM at pH 8.0, indicating that palythine and porphyra-334 had a great potential for antioxidant materials in red alga. On the other hand, these MAAs exhibited a low activity at low pH. ABTS scavenging acitivity of palythine (72 µM) at pH 5.8 and 6.6 was 0.8 and 24.8%, respectively, and that of porphyra-334 (72 µM) at pH 5.8 and 6.6 was 10.0 and 21.0%, respectively.

### 2.4. Ferrous Reducing Power of Dulse MAAs

In addition to ABTS radical scavenging activity, ferrous reducing power was also evaluated in the different pH. Ferrous reducing power assay is based on Reactions 2 and 3 (MAA• represents MAA radicals) [37,38]:

Fe(CN)_6_^3−^ + MAA-H → Fe(CN)_6_^4−^ + MAA^•^ + H^+^ (2)

Fe(CN)_6_^4−^ + Fe^3+^ + K^+^ → KFe[Fe(CN)_6_] (3)

**Figure 5:**
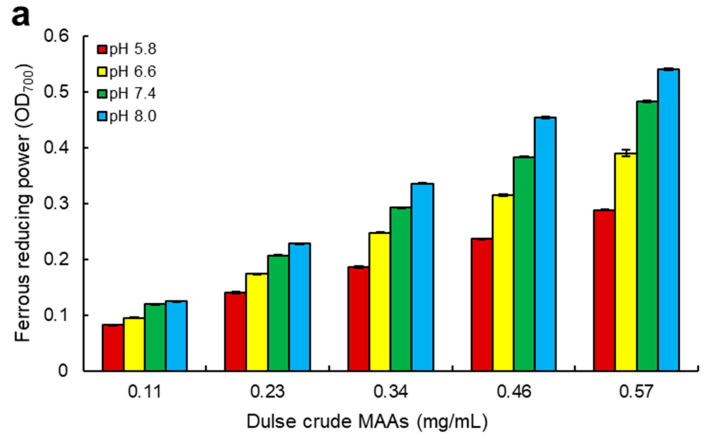

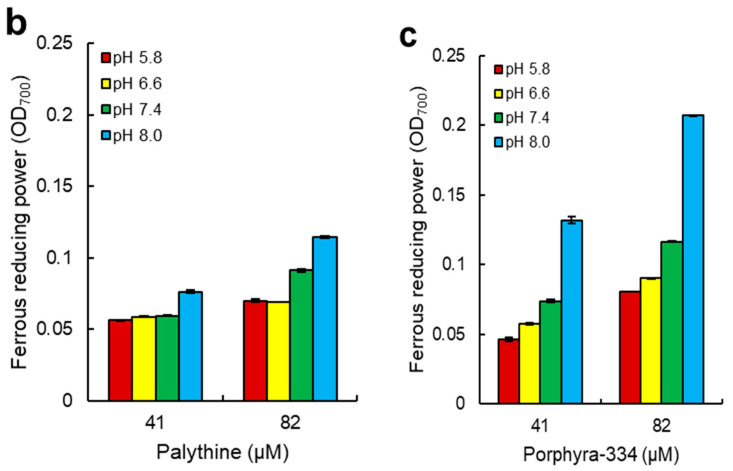
Ferrous reducing power assay. (**a**), dulse crude MAAs; (**b**), palythine; (**c**), porphyra-334. Reducing power assay was performed at pH 5.8, 6.6, 7.4 and 8.0. Data represent mean values ± SE (*n* = 3). The components of dulse crude MAAs in 0.11 mg/mL were as follows: shinorine (0.22 µM), palythine (1.8 µM), asterina-330 (0.07 µM), porphyra-334 (2.3 µM), and usujirene + palythene (0.95 µM).

Ferrous reducing power of dulse in Usujiri was determined using dulse crude MAAs, palythine and porphyra-334, resulting in the exhibition of ferrous reducing power in samples (Figure 5). The ferrous reducing power was also increased in the alkaline condition, and the maximum ferrous reducing power was at pH 8.0. The content of dulse crude MAAs, which reached the value of OD_700_ shows 0.10 (MC_700_ 0.10) at pH 5.8, 6.6, 7.4, and 8.0, were 0.17, 0.13, 0.11, and 0.10 mg/mL, respectively (Table 5). Palythine and porphyra-334 also showed the highest ferrous reducing power at alkaline condition (pH 8.0) with the MC_700_0.10 values of 66.9 and 36.2 µM, respectively. Interestingly, ferrous reducing power of palythine was lower than that of porphyra-334, whereas ABTS scavenging activity of palythin was higher than that of porphyra-334. Comparing the MC_700_0.10 values of these MAAs with ascorbic acid, ferrous reducing power of MAAs was more than 70-fold lower, showing that the difference in the antioxidant capacity between ascorbic acid and MAAs was larger in ferrous reducing power than that in ABTS radical scavenging activity. Therefore, it was revealed that the radical scavenging activity was important in the antioxidant capacity of MAAs.

## 3. Discussion

### 3.1. Efficient Extraction of MAAs from Dulse

In the present study, we determined the efficient extraction condition of MAAs and investigated the monthly variation (January–May 2019) of Usujiri dulse. The present method has the advantage in the content and purity of MAAs. Many studies used methanol only or 50% methanol for extraction solution. Therefore, we compared the content of MAAs by water extraction and methanol only extraction, showing that the content by water extraction was approximately two times higher than that by methanol extraction (Table 6). However, the water extraction contained many proteins and salts. Therefore, we applied methanol only extraction to remove above impurities, resulting in the purity of samples being sufficient for HPLC systems. As a result, 6 h water extraction was suitable, and sample collected at the end of February contained a high content of MAAs. The detected six MAAs were increased from 2 to 6 h water extraction. However, five MAAs were decreased from 6 to 24 h water extraction except for palythine. Usujirene and palythene are unstable in acidic condition, yielding palythine by hydrolysis of cis-trans isomerization form [39,40,41]. Comparing the spectra of 6 and 24 h (Figure 1), the absorbance (357–360 nm) showing the absorption maxima of usujirene and palythene was significantly decreased. In addition, the decrease in usujirene + palythene and increase in palythine from 6 to 24 h were close (Table 2). The increase in palythine would be related to the hydrolysis of usujirene and palythene. On the other hand, the content of shinorine and porphyra-334 was significantly decreased (*p* < 0.05) from 6 to 24 h. Porphyra-334 was stable in solutions with the broad pH from 1 to 11 for 24 h at room temperature [42]. Sinha et al. [43] reported that shinorine and porphyra-334 were stable at 75 °C for up to 6 h. Coba et al. [44] reported that MAAs were highly stable at room temperature during 24 h at pH 4.5–8.5 at 50 °C. Chaves-Peña et al. [45] reported that the content of MAAs by water extraction and 20% methanol extraction was stable. When we extracted MAAs using methanol only, the content of the MAAs was stable despite of the extraction term (Table 6). Thus, we hypothesized the decrease in shinorine and porphyra-334 comes from enzymatic hydrolysis from dulse components.

**Figure 6:**
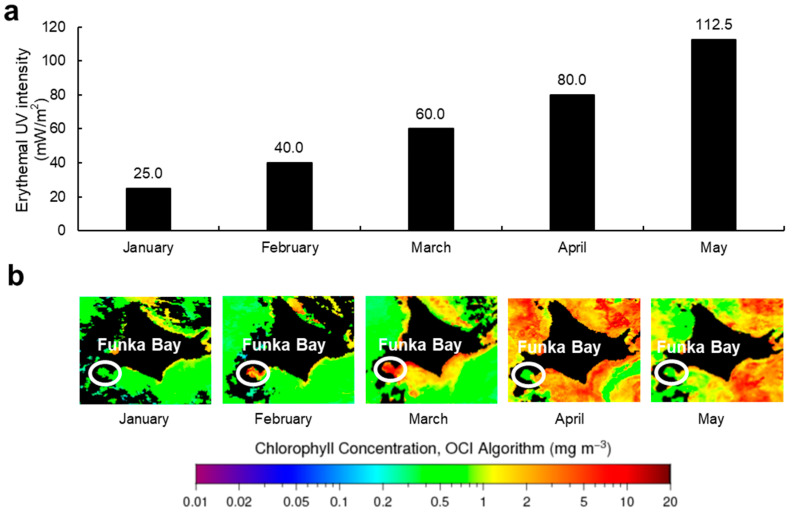
The monthly mean of daily maximum erythemal UV intensity and chlorophyll concentration. (**a**) erythemal UV intensity; (**b**), chlorophyll concentration. These data were obtained from JMA and NASA’s Ocean Color WEB, respectively. All data were recorded in 2019.

The content of MAAs in Usujiri dulse was varied from 2.649 to 6.930 µmol (from 0.735 to 2.081 mg) g^−1^ DW. On the other hand, the content of MAAs in *P*. *palmata* from Galway Bay (Ireland) was from 0.620 on September to 7.340 mg g^−1^ DW on April [46] revealed that the content of MAAs in dulse was varied and that of Usujiri was low. One of the factors affected the content of MAAs is related to seasonal variation. It was reported that the content of MAAs in red algae *Nothogenia fastigiata* and *Corallina officinalis* collected from Bahía Mansa was increased in the high solar radiation (spring and summer season) [9]. Correlation of MAAs and solar radiation was reported in the red seaweed *Gracilaria vermiculophylla* cultured outdoors, the planktonic organisms from lakes and red algae *P. palmata* and *Devaleraea ramentacea* from the Kongsfjorden (Spitsbergen) and *P*. *palmata* from Galway Bay (Ireland) [46,47,48,49,50]. Although UVR and erythemal UV show mainly UVA and UVB, respectively, erythemal UV intensity is a good parameter for the representation of UVR. Therefore, we obtained erythemal UV intensity data of Hakodate in 2019 from Japan Meteorological Agency (accessed on 24 August 2020) (JMA: https://www.data.jma.go.jp/gmd/env/uvhp/info_uv.html) (Figure 6a). The monthly mean of maximum erythemal UV intensity in Hakodate gradually increased from January (25.0 mW/m^2^) to May (112.5 mW/m^2^). Unfortunately, correlation between MAAs and erythemal UV intensity was not found. Erythemal UV intensity was increased from January to May; however, the content of MAAs was not. We thought that the difference would come from the environmental reason such as lack of nutrients, water temperature, desiccation and thermal stress [9,13,39,51].

Nutrient availability (e.g., nitrogen) also affected the content of MAAs in red algae [9,18,52,53,54]. Usujiri is located at the entrance of Funka Bay. In Funka Bay, the large proliferation of diatoms is observed every spring (spring bloom). NASA’s Ocean Color WEB site provided chlorophyll concentration of the world (https://oceancolor.gsfc.nasa.gov) (accessed on 24 August 2020). Usually, chlorophyll concentration was related to the increase in diatoms and the decrease in the dissolved inorganic nitrogen in the surface layer. To investigate the relationship between chlorophyll concentration and the content of MAAs in Usujiri dulse, we prepared chlorophyll concentration data in 2019 (Figure 6b). The chlorophyll concentration in Funka Bay was increased sharply from January (below 1.0 mg/m^3^) to March (below 20 mg/m^3^), decreased sharply March to April (below 1.0 mg/m^3^) and stable from April to May (below 1.0 mg/m^3^). Thus, it was clear that spring bloom peak in Funka Bay in 2019 was reached in March, suggesting that the decrease of nitrogen supply from seawater was one of the key factors affected the content of MAAs in Usujiri dulse. Comparing the content of MAAs in Usujiri and Galway Bay (Ireland), Usujiri dulse has more potential to accumulate MAAs. We thought that a multi-year investigation is necessary to clarify the relationship between the accumulation of MAAs in Usujiri dulse and environmental conditions.

Palythine and porphyra-334 were major MAAs in Usujiri dulse. Karsten et al. [55] showed that *P*. *palmata*, which grew in a depth of 4 or 10 m, synthesized the equivalent content of shinorine, porphyra-334, and palythine, while the alga that grew at a depth of 2 m synthesized more palythine. We collected Usujiri dulse in the depth of 1 m, suggesting that the high molar percentage of palythine in Usujiri dulse may be related to the water depth. Other characteristic MAAs in Usujiri dulse are the high molar percentage of usujirene + palythene. Usujirene and palythene can absorb strong UVA due to the presence of cis-trans double bond in the structure [23,37,40]. Furthermore, hypothetic pathways for biosynthesis of MAAs showed that porphyra-334 is converted to usujirene [18,39,56]. Athukorala et al. [57] reported that red alga *Mastocarpus stellatus* was converted porphyra-334 to usujirene with an increase in the UVA exposure. The mol% of usujirene + palythene in Usujiri dulse was increased with erythemal UV intensity from January to April in 2019 (Figure 3 and Figure 6a). Thus, it is likely that porphyra-334 in Usujiri dulse was also converted to usujirene or palythene with an increase in UVA exposure.

### 3.2. Antioxidant Capacity of MAAs from Dulse

Despite many studies, research on the capacity related to pH was limited. In the present study, it was revealed that dulse crude MAAs, palythine, and porphyra-334 showed the highest scavenging activity and reducing power at alkaline condition in two ET reaction assays (Figure 4 and Figure 5). To our knowledge, this is the first report on the relationship of antioxidant capacity between pH and palythine.

In the ABTS radical scavenging assay, the components of dulse crude MAAs in 0.15 mg/mL were as follows: shinorine (0.3 µM), palythine (2.4 µM), asterina-330 (0.1 µM), porphyra-334 (3.2 µM), and usujirene + palythene (1.3 µM), showing that a total of 7.3 µM MAAs was contained. Although usujirene is a strong antioxidant MAA [23,58,59], the main content of MAAs in Usujiri dulse was palythine and porphyra-334. We applied the antioxidant capacity of the purified MAAs to that of dulse crude MAAs, showing that two MAAs covered approximately 50% of ABTS radical scavenging activity at pH 7.4. On the other hand, the ratio of ABTS radical scavenging activity of MAAs at pH 8.0 was only 25%, indicating that antioxidant substances except for MAAs would be involved in alkali conditions. It is reported that amino acids and phenolic compounds are also known as pH-dependent antioxidants [58,59,60,61]. Phenolic compounds showed the significantly stronger antioxidant capacity than imino-MAAs (shinorine, porphyra-334, palythine, asterina-330 and palythinol) [10]. When we applied the ferrous reducing power of the two purified MAAs to that of dulse crude MAAs, the ratio of the purified MAAs was low (>5%) in the tested pH conditions. Therefore, it was suggested that MAAs were the key antioxidant materials at neutral pH in red algae, and MAAs showed the strong antioxidant capacity in the electron transfer reaction (radical scavenging activity).

**Figure 7:**
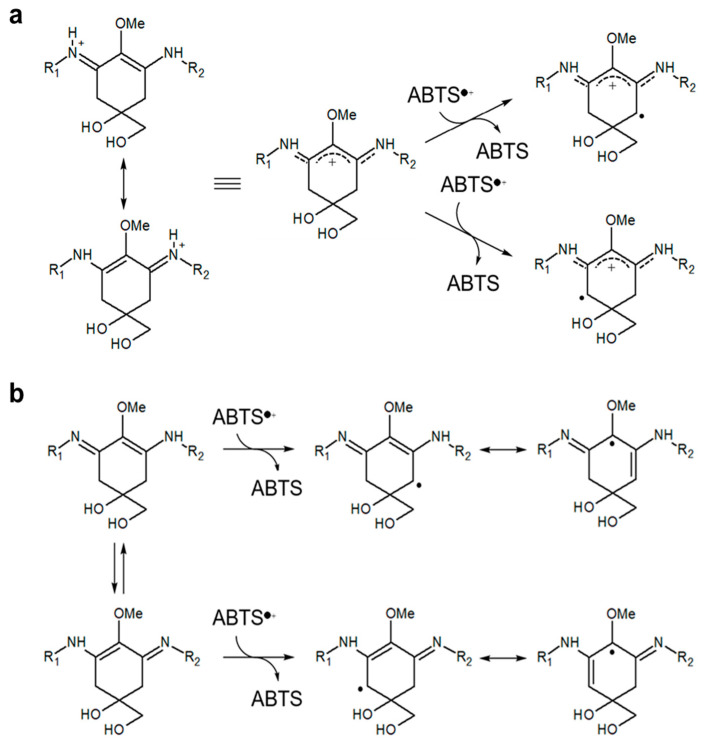
Putative radical stabilization mechanism of MAAs. Stabilization of ABTS radical was occurred by resonance of radical electron at the C-4 and C-6 position in imino-MAAs. (**a**) acidic condition; (**b**) basic condition.

Torres et al. [10] demonstrated that high antioxidant capacity of shinorine and porphyra-334 was found in alkaline conditions (pH 9.6). De La Coba et al. [21] reported that MAAs were pH-dependent antioxidants. These authors showed that the IC_50_ values of MAAs were decreased approximately 6 to 100-fold by the change of pH 6.0 to pH 8.5. Apak et al. [37] pointed out that the alkaline condition may promote the deprotonation of susceptible molecules and concomitantly increase their electron transfer capacity. When hydrogen atoms are abstracted from C-4 and/or C-6 position of methylene, the radical electron is delocalized in cyclohexene groups. Although this delocalization stabilizes the radical species formed, pH conditions changed the delocalization form of MAAs. The radical electrons of imino-MAAs were already delocalized at high pH, resulting in the increase of reactivity (Figure 7b) [38,58]. When imino-MAAs were protonated at low pH, the radical electron cannot widely delocalize, resulting in the decrease of reactivity (Figure 7a). The reactivity showed a good relationship between delocalization and the kinetics of antioxidant capacity of MAAs [10].

Ascorbic acid, an excellent one-electron reducing reagent, exerts pro-oxidant effects in the presence of catalytic metal ions at neutral conditions [62,63,64,65], while ascorbate monoanion (ASH^−^) generates the strong hydroxyl radical (HO•) through multiple reactions. Namely, ASH^-^ reduces ferric (Fe^3+^) to ferrous (Fe^2+^) iron (Reaction 4). Fe^2+^ readily reacts with O_2_ and produces superoxide radical (O_2_^•−^) (Reaction 5), resulting in the production of H_2_O_2_ (Reaction 6). Finally, Fe^2+^ reacts with H_2_O_2_ generating HO^•^ (Reaction 7) in a classic Fenton reaction, which is the reactive and harmful ROS for living body. On the other hand, MAAs showed the low reducing power and moderate radical scavenging activity at neutral conditions (Table 4 and Table 5), suggesting that MAAs might be the superior antioxidants materials in terms of the safety:

AscH^−^ + Fe^3+^ → Asc^•−^ + Fe^2+^ (4)

Fe^2+^ + O_2_ → Fe^3+^ + O_2_^•−^ (5)

O_2_^•−^ + O_2_^•−^ + 2H^+^ → H_2_O_2_ + O_2_ (6)

Fe^2+^ + H_2_O_2_ → Fe^3+^ + HO^•^ (Fenton reaction) (7)

## 4. Materials and Methods

### 4.1. Algal Material

All dulse samples were collected at 1 m depth in Usujiri, Hakodate, Japan (41°56′N, 40°56′E) on March 2018 and from January to May 2019. After collection, the thalli were washed with tap-water to remove sea salt and epibionts. Soon after, they were lyophilized. Dried algal samples were ground into a fine powder by Wonder Blender WB-1 (Osaka Chemical Co., Osaka, Japan) and stored in the dark at room temperature until analysis.

### 4.2. Determination of the Extraction Condition of Dulse Crude MAAs

To determine the optimum extraction conditions of MAAs, water extraction time from dulse powder was evaluated. The powdered samples were soaked in 20 volumes (*v*/*w*) of distilled water at 4 °C for 2 to 24 h. The water extracts were collected by centrifugation at 4 °C, 27,200 g for 10 min. After centrifugation, the supernatants were lyophilized and soaked in 20 volumes (volume/powdered sample weight) of methanol at 4 °C for 2 h. The MAAs containing methanol extracts were centrifuged at 4 °C, 27,200× *g* for 15 min, and designated as dulse crude MAAs solutions. The supernatants were evaporated, re-dissolved in water, and lyophilized. Then, the solid samples were designated as dulse crude MAAs and used following experiments.

### 4.3. Spectrophotometric Analysis of Dulse Crude MAAs Solutions

Dulse crude MAAs solutions were analyzed by the UV-visible ray absorption spectrum using a spectrophotometer (250–400 nm, UV-1800, Shimadzu, Kyoto, Japan).

### 4.4. Separation of MAAs by HPLC

The dulse crude MAAs were dissolved in ultra pure water containing 0.1% trifluoroacetic acid (TFA) and applied to sequential filtration by Millex-GV (pore size: 0.22 μm) (Merck Millipore, Billerica, MA, USA) and Millex-LG (pore size: 0.20 μm) (Merck Millipore). The filtrated MAAs were isolated by reversed-phase HPLC with a Mightysil RP-18GP column (5 µm, 10 × 250 mm) (Kanto Kagaku, Tokyo, Japan) using an isocratic elution of ultra pure water containing 0.1% TFA for 7 min and a linear gradient of acetonitrile (0–70%) containing 0.1% TFA for 13 min at a flow rate of 4.73 mL/min. The column oven temperature was set at 40 °C. The detection wavelength was set at 330 nm. The peaks having 330 nm were fractionated and evaporated. Then, the purified MAAs were dissolved in an appropriate amount of distilled water.

### 4.5. Identification of MAAs by MALDI-TOF/MS

The mass-to-charge ratio of MAAs was determined by the Matrix Assisted Laser Desorption/Ionization Time Of Flight Tandem Mass Spectrometry (MALDI-TOF/MS) method using a 4700 Proteomics Analyzer with Denovo Explorer software (Applied Biosystems, Carlsbad, CA, USA). The fractionated MAAs were lyophilized and dissolved in ultra pure water containing 0.1% TFA. Then, the samples were mixed with 5 mg/mL α-cyano-4-hydroxycinnamic acid matrix and detected by positive-mode.

### 4.6. Calculation of the Content of MAAs in HPLC

The content of individual MAAs was determined using Lambert–Beer law. Using the purified individual MAAs, the relationship between the content of MAAs and HPLC peak area was determined. The results were expressed as µmol g^−1^ DW (dry weight).

### 4.7. ABTS Radical Scavenging Assay

ABTS radical scavenging assay was carried out according to the method of Binsan et al. [66] with some modifications. The working solution was prepared by mixing with an equal volume of 14.8 mM ABTS and 5.2 mM potassium persulphate and incubated at room temperature in dark conditions for 12 h. The ABTS reagent having the absorbance at 734 nm of 1.00 ± 0.02 was prepared by the dilution of working solution with 0.2 M phosphate buffer (pH 5.8, 6.6, 7.4, and 8.0). The ABTS radical scavenging assay was performed as follows: 50 µL of sample (dulse crude MAAs or the purified MAAs) or distilled water were mixed with 950 µL of the ABTS reagent or the phosphate buffer. Then, the mixture was incubated at room temperature for 2 h in the dark. After the incubation, the solution was centrifuged at 4 °C, 2000× *g* for 5 min to remove insoluble, and the supernatant was measured absorbance at 734 nm. ABTS radical scavenging activity (%) was calculated from the equation [1 − (As − Asb)/(Ac − Acb)] × 100, where As is the absorbance of sample mixed with the ABTS reagent, Asb is the absorbance of sample mixed with the phosphate buffer, Ac is the absorbance of distilled water mixed with the ABTS reagent, and Acb is the absorbance of distilled water mixed with the phosphate buffer. Ascorbic acid was used as a standard.

### 4.8. Ferrous Reducing Power Assay

Ferrous reducing power was determined according to the method of Kuda et al. [67] with some modifications. In addition, 0.4 mL of sample (dulse crude MAAs or the purified MAAs) or distilled water were mixed with 0.4 mL of 0.2 M phosphate buffer (pH 5.8, 6.6, 7.4, and 8.0) and 0.4 mL of 1% potassium ferricyanide. The mixture was incubated at 50 °C for 20 min. Then, 0.4 mL of 10% trichloroacetic acid was added to the reaction mixture. A half volume of samples were extracted and then mixed with 960 µL of 0.017% ferric chloride and incubated for 10 min at room temperature. Absorbance at 700 nm of solutions was measured. Ferrous reducing power (OD) was calculated from the equation As-Asb, where As is the absorbance of sample and Asb is the absorbance of control. Ascorbic acid was used as a standard.

### 4.9. Abiotic Data in Hakodate (Usujiri)

The monthly mean of daily maximum ultraviolet index (UVI) was obtained from Japan Meteorological Agency (JMA: https://www.data.jma.go.jp/gmd/env/uvhp/info_uv.html). According to the method of JMA, erythemal UV intensity (mW/m^2^) was calculated by multiplying UVI by 25 times. Data on chlorophyll concentration (mg/m^3^) of near-surface were obtained from NASA’s Ocean Color WEB (https://oceancolor.gsfc.nasa.gov). All data were recorded in 2019.

### 4.10. Statistical Analysis

Data are expressed as the mean ± standard error. All values are mean of triplicate analysis. Statistical analysis was carried out using Tukey–Kramer’s multiple comparisons test. All statistical analyses were performed using Statcel 3 software (Version No. 3, OMS Publisher, Tokorozawa, Japan).

## 5. Conclusions

In the present study, we determined the efficient extraction condition of MAAs and then investigated the monthly variation (January–May 2019) in the content of MAAs in Usujiri dulse. As a result, it was revealed that a large content of MAAs were obtained by water extraction for 6 h from Usujiri dulse collected in late February. We hypothesized that not only solar radiation, but also chlorophyll concentration affected monthly variation in the content of MAAs in Usujiri dulse. It is necessary to conduct a multi-year investigation to validate this hypothesis. Finally, we evaluated the effects of pH on the antioxidant capacity of MAAs from Usujiri dulse by ABTS radical scavenging and ferrous reducing power assays. As a result, it was revealed that dulse crude MAAs, palythine, and porphyra-334 showed the highest scavenging activity and reducing power in alkaline conditions in two ET reaction assays. The pH of our skin is acidic, meaning that antioxidant capacity of MAAs does not show the good potential. On the other hand, the pH of some part of our body is weak alkali such as eye and blood, indicating that MAAs will be good sunscreen and antioxidant materials. This suggests that pH must be considered when using MAAs as antioxidant components in biotechnology products.

## Figures and Tables

**Table 1:** The chemical formula of MAAs.

Peak No. ^1^	MAAs	Formula	Extinction Coefficient ^2^ (M^−1^ cm^−1^)	Retention Time ^3^ (min)	λ_max_^4^ (nm)	*m*/*z*^5^ [M + H]^+^
a	Shinorine	C_13_H_20_N_2_O_8_	44,700	4.42	329–331	333.2
b	Palythine	C_10_H_16_N_2_O_5_	36,200	4.79	319	245.1
c	Asterina-330	C_12_H_20_N_2_O_6_	43,500	5.27	325–328	289.2
d	Porphyra-334	C_14_H_22_N_2_O_8_	42,300	7.73	331	347.1
e	Usujirene	C_13_H_20_N_2_O_5_	45,070	13.36	357	285.1
f	Palythene	C_13_H_20_N_2_O_5_	47,521	13.43	358	285.1

^1^ Data from Figure 2. ^2^ Data from [34] (shinorine, palythine and asterina-330), [35] (porphyra-334) and [36] (usujirene and palythene).^3^ Data from HPLC.^4^ Data from UV-Vis-absorption. ^5^ Data from MALDI-TOF/MS.

**Table 2:** The content of extracted MAAs from dulse at different water extraction term.

MAAs	Water Extraction Term		
	2 h	6 h	24 h
Shinorine	0.147 ± 0.003 ^a^	0.155 ± 0.001 ^a^	0.137 ± 0.002 ^b^
Palythine	2.519 ± 0.140 ^b^	2.964 ± 0.032 ^a^	3.130 ± 0.049 ^a^
Asterina-330	0.069 ± 0.002 ^b^	0.078 ± 0.001 ^a^	0.074 ± 0.001 ^a b^
Porphyra-334	1.771 ± 0.013 ^b^	1.900 ± 0.029 ^a^	1.688 ± 0.020 ^b^
Usujirene + Palythene	0.258 ± 0.007 ^a^	0.276 ± 0.031 ^a^	0.131 ± 0.006 ^b^
Total	4.764 ± 0.161 ^b^	5.372 ± 0.066 ^a^	5.160 ± 0.068 ^a^

The content of MAAs at each water extraction term (2, 6, and 24 h) was expressed as µmol g^−1^ dry weight (DW). The data show mean values ± SE, *n* = 3. Difference letters in each MAA indicate significant differences in mean value (Tukey–Kramer’s multiple comparisons test, ^a, b^ *p* < 0.05).

**Table 3:** The content of MAAs in Usujiri dulse.

MAAs	Collection Date (2019)			
	January 23	February 25	April 2	May 17
Shinorine	0.073 ± 0.001 ^d^	0.266 ± 0.002 ^a^	0.166 ± 0.001 ^b^	0.089 ± 0.001 ^c^
Palythine	1.739 ± 0.011 ^d^	2.289 ± 0.017 ^b^	2.112 ± 0.058 ^c^	3.255 ± 0.007 ^a^
Asterina-330	0.035 ± 0.000 ^d^	0.097 ± 0.002 ^b^	0.118 ± 0.001 ^a^	0.084 ± 0.000 ^c^
Porphyra-334	0.778 ± 0.009 ^d^	3.083 ± 0.034 ^a^	2.507 ± 0.015 ^b^	0.972 ± 0.001 ^c^
Usujirene + Palythene	0.024 ± 0.002 ^d^	1.194 ± 0.009 ^b^	1.720 ± 0.034 ^a^	0.572 ± 0.006 ^c^
Total	2.649 ± 0.020 ^d^	6.930 ± 0.045 ^a^	6.623 ± 0.032 ^b^	4.972 ± 0.004 ^c^

Dulse was collected on January 23, February 25, April 2, and May 17, 2019. The content of MAAs is expressed as µmol g^−1^ DW. The data show mean values ± SE, *n* = 3. Difference letters in each MAA indicate significant differences in mean value (Tukey–Kramer’s multiple comparisons test, ^a, b, c, d^ *p* < 0.05).

**Table 4:** IC_50_ value of ABTS radical scavenging assay of MAAs.

Compounds	pH							
	5.8		6.6		7.4		8.0	
	µM	µg/mL	µM	µg/mL	µM	µg/mL	µM	µg/mL
Dulse crude MAAs	―	360	―	330	―	280	―	140
Palythine	>72.0	>18	>72.0	>18	23.4	5.7	12.0	2.9
Porphyra-334	>72.0	>25	>72.0	>25	27.5	9.5	20.8	7.2
Ascorbic acid	19.1	3.4	19.4	3.4	12.4	2.2	8.9	1.6

IC_50_ value represents as µM and µg/mL. Values are expressed as means of triplicate measurements (*n* = 3). ―, Not Calculated.

**Table 5:** Ferrous reducing power assay of MAAs.

Compounds	pH							
	5.8		6.6		7.4		8.0	
	µM	µg/mL	µM	µg/mL	µM	µg/mL	µM	µg/mL
Dulse crude MAAs	―	170	―	130	―	110	―	100
Palythine	>82.0	>2.0	>82.0	>2.0	>82.0	>2.0	66.9	16.0
Porphyra-334	>82.0	>2.0	>82.0	>2.0	67.7	23.0	36.2	13.0
Ascorbic acid	6.0	1.1	3.9	0.69	2.1	0.37	0.5	0.088

The value represents the content of MAAs (µM and µg/mL) reached OD_700_ = 0.10. Values are expressed as means of triplicate measurements (*n* = 3). ―, Not Calculated.

**Table 6:** The content of extracted MAAs from dulse at different methanol only extraction term.

MAAs	Methanol Only Extraction Term		
	2 h	6 h	24 h
Shinorine	0.056 ± 0.006 ^a^	0.062 ± 0.000 ^a^	0.062 ± 0.000 ^a^
Palythine	1.428 ± 0.010 ^a^	1.357 ± 0.009 ^b^	1.366 ± 0.008 ^b^
Asterina-330	0.032 ± 0.001 ^a^	0.031 ± 0.000 ^a^	0.031 ± 0.000 ^a^
Porphyra-334	0.787 ± 0.002 ^a^	0.766 ± 0.007 ^a^	0.755 ± 0.004 ^b^
Usujirene + Palythene	0.127 ± 0.012 ^a^	0.122 ± 0.007 ^a^	0.114 ± 0.007 ^a^
Total	2.429 ± 0.014 ^a^	2.329 ± 0.021 ^b^	2.327 ± 0.017 ^b^

The content of MAAs at each methanol extraction term (2, 6, and 24 h) was expressed as µmol g^−1^ dry weight (DW). The data show mean values ± SE, *n* = 3. Difference letters in each MAA indicate significant differences in mean value (Tukey–Kramer’s multiple comparisons test, ^a, b^ *p* < 0.05).
